# Supplementary material for: Impact of Using Oilseed Industry Byproducts Rich in Linoleic and Alpha-Linolenic Acid in Ruminant Nutrition on Milk Production and Milk Fatty Acid Profile
Source: Animals (Basel). 2024 Feb 6;14(4):539. doi: 10.3390/ani14040539 (PMC10886360; doi:10.3390/ani14040539)
Supplement: Supplementary file 1 [file animals-14-00539-s001.zip › animals-2756188-supplementary.pdf]

Supplementary Table S1. Dietary ingredients and chemical composition of the control and experimental diets.

**Cow - Hempseed cake [35]**				
	Control diet	Experimental diet		
DMI (kg/day)	23.3	26.4	23.9	26.4
Ingredient (g/kg DM)				
Grass silage	499	495	493	490
Compound pellets (barley, oats, sugar beet pulp, wheat bran, molasses, vitamins and minerals)	501	362	274	192
Hempseed cake	-	143	233	318
Chemical composition (g/kg DM if not otherwise stated)				
DM (g/kg)	451	455	457	459
Crude protein	126	157	177	195
Crude fat	23	37	46	54
Ash	70	70	70	71
NDF	346	371	386	401
ADF	210	240	258	276
Starch	228	172	136	103
BSN (buffer soluble N) (g/kg N)	383	339	319	305
NPN (non-protein N)(g/kg N)	337	281	255	236
ADIN (acid detergent insoluble N) (g/kg N)	41	52	57	60
ME (MJ/kg DM)	11.7	11.4	11.2	11.0

**Sheep - Hempseed cake [36]**		
	Control diet	Experimental diet
DMI (kg/day)	1.92	1.95
Ingredient (kg/d per animal, as fed)		
Grass hay	0.900	0.900
Alfalfa hay	0.500	0.500
Corn	0.35	0.23
Barley	0.13	0.05
Sunflower meal	0.34	0.06
Hempseed cake	-	0.48
Mineral mix	0.03	0.03
Chemical composition (% DM if not otherwise stated)		
DM (%)	89.01	89.27
Crude protein	16.12	16.03
Ether extract	2.21	4.50
NDF	38.41	44.20
ADF	22.80	25.02
NE_L (Mcal/kg DM)	1.327	1.330

**Goat – Hempseed cake [37]**			
	Control diet	Experimental diet	
DMI (kg/day)	nr	nr	nr
Ingredient (g/kg) in concentrate			
Corn	500	500	500
Barley	132	132	132
Wheat flour	100	100	100
Hempseed cake	-	60	120
Soybean meal (46% CP)	100	80	70
Extruded soybean	140	100	50
Calcium carbonate	13	13	13
Mineral vitamin premix	10	10	10
Chemical composition (g/kg DM if not otherwise stated) of concentrate			
DM (g/kg)	881	892	893
Crude protein	165	163	162

Crude fibre	40.9	52.6	73.4
Crude ash	50.0	49.1	49.3
Crude fat	55.7	53.4	46.7
ME (MJ/kg DM)	12.4	12.3	12.2
NDF	43.1	37.9	30.2
ADF	8.14	9.04	11.7
ADL	3.12	2.45	4.28
N-free extract	689	682	669
**Cow – Pumpkin seed cake [40]**			
	Control diet	Experimental diet	
DMI (kg/day)	20.1	19.7	19.7
Ingredient (% DM)			
Alfalfa hay	22.0	22.0	22.0
Corn silage	27.7	27.7	27.7
Ground corn	29.4	29.4	29.4
Cottonseed meal	4.9	4.9	4.9
Soybean meal	13.2	6.6	-
Pumpkin seed cake	-	4.4	8.3
DDGS	-	2.2	4.9
Premix	2.8	2.8	2.8
Chemical composition (% DM if not otherwise stated)			
Crude protein	16.3	16.2	17.0
NDF	37.0	35.6	36.7
ADF	22.0	22.0	22.6
Starch	24.3	24.2	24.2
Ether extract	2.75	3.07	3.35
NE_L (Mcal/kg DM)	1.64	1.64	1.64
**Cow – Pumpkin seed cake [41]**			
	Control diet	Experimental diet	
DMI (kg/day)	24.97	24.67	24.43
Ingredient (% DM)			
Alfalfa hay	18.67	18.67	18.67
Corn silage	29.78	29.78	29.78
Ground corn	27.10	27.10	27.10
Cottonseed meal	3.56	3.56	3.56
Soybean meal	17.78	8.89	-
High-oil pumpkin seed cake	-	5.56	12.00
DDGS	-	3.33	5.78
Premix	2.22	2.22	2.22
Megalac	0.89	0.89	0.89
Chemical composition (% DM if not otherwise stated)			
Crude protein	17.00	17.00	17.16
RDP (% of CP)	62.85	62.98	63.11
RUP (% of CP)	37.15	37.02	36.89
NDF	27.74	28.85	29.91
ADF	17.04	17.59	18.01
Starch	27.02	26.93	26.75
Ether extract	3.43	4.17	5.00
Forage NDF	21.74	21.74	21.74
Physically effective NDF	22.31	22.29	22.33
NE_L (Mcal/kg DM)	1.71	1.71	1.72
ME for milk (kg/day)	41.8	42.1	42.4
MP for milk (kg/day)	40.0	41.8	43.9
**Goat - Pumpkin seed cake [38]**			
	Control diet	Experimental diet	
DMI (kg/day)	1.92	1.91	
Ingredient (%) in concentrate			

Corn grains	54.90	48.40
Barley	29.00	37.10
Sunflower meal	13.70	-
Pumpkin seed cake	-	12.10
Monocalcium phosphate	0.80	0.80
Calcium carbonate	0.80	0.80
Salt	0.80	0.80
Nutritional content of the diets		
NE_L (UFL/kg DM/day)	1.81	1.81
PDIN (protein truly digested in the small intestine when the protein is the limiting factor) (g/day)	167.90	167.60
PDIE (protein truly digested in the small intestine when the energy is the limiting factor) (g/day)	187.40	181.40
Ether extract (g/day)	43.49	61.30
**Goat - Pumpkin seed cake [39]**		
	Control diet	Experimental diet
DMI (kg/day)	nr	nr
Ingredient (g/kg as fed) in concentrate		
Corn	429	459
Barley	80	90
Oats	100	135
Wheat flour	120	120
Extruded soybean meal	150	-
Pumpkin seed cake	-	160
Soybean meal (46% CP)	85	-
Calcium carbonate	16	16
Monocalcium phosphate	5	5
Salt	4	4
Pellet binder	1	1
Premix	10	10
Chemical composition (g/kg DM if not otherwise stated) of concentrate		
DM (g/kg fresh matter)	876	873
Crude protein	162	163
Crude fibre	41.4	37.3
Crude ash	49.2	52.3
Crude fat	56.4	56.3
ME (MJ/kg DM)	13.2	13.2
DOM (digestibility of organic matter)	819	822
**Goat - Pumpkin seed cake [65]**		
	Control diet	Experimental diet
DMI (kg/day)	nr	nr
Ingredient (g/kg as fed) in concentrate		
Corn	429	459
Barley	80	90
Oats	100	135
Wheat flour	120	120
Extruded soybean meal	150	-
Pumpkin seed cake	-	160
Soybean meal (46% CP)	85	-
Calcium carbonate	16	16
Monocalcium phosphate	5	5
Salt	4	4
Pellet binder	1	1
Premix	10	10
Chemical composition (g/kg DM if not otherwise stated) of concentrate		

DM (g/kg fresh matter)	876		873	
Crude protein	162		163	
Crude fibre	41.4		37.3	
Crude ash	49.2		52.3	
Crude fat	56.4		56.3	
NFE (nitrogen free extract)	691		691	
ME (MJ/kg DM)	13.2		13.2	
**Cow – Sunflower meal [24]**				
	Control diet	Experimental diet		
DMI (kg/day)	22.0	21.4	21.2	21.9
Ingredient (g/kg DM)				
Maize silage	550	550	550	550
Ground maize grain	211	211	211	211
Soybean meal	112.5	75.0	37.5	-
Wheat middlings	97.5	65.0	32.5	-
Sunflower meal	-	70.0	140	210
Urea : ammonium sulphate (9:1)	10.0	10.0	10.0	10.0
Limestone	9.7	9.7	9.7	9.7
Dicalcium phosphate	3.0	3.0	3.0	3.0
Salt	4.8	4.8	4.8	4.8
Mineral premix	1.5	1.5	1.5	1.5
Chemical composition (g/kg DM if not otherwise stated)				
DM (g/kg)	414	414	414	415
Organic matter	959	960	961	962
Crude protein	162	162	162	163
Non protein nitrogen (g/kg N)	458	470	483	495
Neutral detergent insoluble nitrogen (g/kg N)	129	133	138	142
Acid detergent insoluble nitrogen (g/kg N)	65.8	65.7	65.5	65.3
Rumen degradable protein	107	109	111	113
Ether extract	23.8	23.1	22.4	21.6
Non-fiber carbohydrate	410	402	394	386
ADF	229	240	251	262
NE_L (MJ/kg DM)	6.04	5.94	5.93	5.70
**Cow – Sunflower meal [66]**				
	Control diet		Experimental diet	
DMI (kg/day)	17.03		17.59	
Ingredient (g/kg DM) in concentrate				
Ground corn	875.5		833.6	
Soybean meal	84.5		-	
Sunflower meal	-		125.8	
Mineral	40.0		40.6	
Chemical composition (g/kg DM if not otherwise stated) of concentrate				
DM (g/kg)	895.3		896.8	
Crude protein	123.6		121.9	
Ether extract	26.5		32.1	
NDF	152.6		173.4	
Indigestible NDF (g/kg NDF)	119.9		321.8	
ADF	48.9		53.5	
Lignin	15.2		17.0	
Neutral insoluble detergent nitrogen	1.8		1.4	
Non-fiber carbohydrate	615.4		592.4	
Ash	81.9		80.2	
Total digestible nutrients	690.6		692.1	
ME (MJ/kg DM)	10.17		10.21	
**Cow – Sunflower meal [72]**				
	Control diet	Experimental diet		

DMI (kg/day)	9.87	9.78	9.89
Ingredient (g/kg as fed)			
Maize	300	300	300
Wheat bran	420	310	190
Groundnut cake	130	130	100
Mustard cake	120	40	-
Sunflower meal	-	190	380
Mineral mixture	20	20	20
Common salt	10	10	10
Chemical composition (g/kg DM if not otherwise stated)			
Organic matter	892.2	876.4	866.1
Crude protein	219.3	222.0	225.0
Ether extract	30.3	23.7	17.6
Total ash	107.8	123.6	133.9
NDF	250.4	272.9	326.9
ADF	123.0	132.2	159.1
ME (MJ/kg)	11.54	11.25	10.93

**Cow – Sunflower meal [50]**		
	Control diet	Experimental diet
DMI (kg/day)	17.8	17.9
Ingredient (%) in concentrate		
Ground shelled corn	48.5	11.5
Rolled oats	27.5	55.6
Soybean meal (50% CP)	22.0	-
Sunflower meal (37% CP)	-	30.9
Dicalcium phosphate	1.0	1.0
Trace mineral salt	1.0	1.0
Chemical composition (% DM if not otherwise stated) of concentrate		
DM (%)	92.9	94.3
Crude protein	19.3	20.0
Ether extract	3.5	3.7
Ash	3.2	4.5
ADF	7.6	14.0

**Cow – Sunflower meal [49]**		
	Control diet	Experimental diet
DMI (kg/day)	17.3	16.9
Ingredient (% DM)		
Sugarcane	60.0	60.0
Ground corn	22.1	14.0
Soybean meal	15.3	3.70
Sunflower meal	-	20.0
Mineral supplement	2.66	2.35
Chemical composition (% DM if not otherwise stated)		
DM (%)	54.7	55.0
Crude protein	13.8	13.4
Ether extract	4.21	4.42
NDFap	33.8	38.1
Indigestible NDF	18.9	23.6
ADF	24.4	29.8
Total carbohydrates	76.9	77.2
Cellulose	19.0	21.9
Hemicellulose	16.9	17.5
Lignin	5.42	7.91
Ash	6.12	5.91
NDIP (neutral detergent insoluble protein)	2.95	4.41
TDN (total digestible nutrients)	67.3	62.4

**Cow – Sunflower meal [68]**

	Control diet	Experimental diet
DMI (kg/day)	20.8	20.5
Ingredient (%) in concentrate		
Maize, ground	12.1	14.2
Maize germ	15.2	15
Wheat, ground	15	15
Barley, ground	14.8	15
Sunflower meal	-	39
Sunflower hulls	8.7	-
Soybean meal	32	-
Limestone	0.75	0.85
Monocalcium phosphate	0.5	-
Common salt	0.7	0.7
Premix	0.25	0.25
Chemical composition (g/kg) of concentrate		
DM	875	883
Crude protein	206.1	207.2
Crude fibre	93.2	93.9
NDF	181.9	158.6
Ether extract	53.2	53.2
Non fibre carbohydrate	427.1	462
**Cow – Sunflower cake [45]**		
	Control diet	Experimental diet
DMI (kg/day)	14.41	14.64
Ingredient (g/kg) in concentrate		
Corn ground	820.6	776.6
Soybean meal	103.4	-
Sunflower cake	-	150.5
Mix minerals	40.6	37.3
Urea + ammonium sulfate (9:1)	35.5	35.7
Chemical composition (g/kg) of concentrate		
DM	958.10	960.30
Ash	56.70	54.90
Crude protein	209.10	197.20
Ether extract	28.70	33.70
NDF	166.00	197.70
ADF	59.00	93.40
Nonfiber carbohydrates	539.50	516.50
Hemicellulose	100.70	104.40
Lignin	1.44	3.05
Total carbohydrates	705.50	714.00
**Cow – Sunflower cake [67]**		
	Control diet	Experimental diet
DMI (kg/day)	20.1	19.9
Ingredient (g/kg DM) in concentrate		
Corn	237	190
Soybean meal	200	150
Cold-pressed sunflower cake	-	230
Palm kernel meal	150	-
Destiled dry grains	149	10
Barley	108	157
Wheat	60	150
Molasses	20	20
Hydrogenated palm fat	20	-
Alfalfa pellets	20	55
Minerals and vitamins	36	38
Chemical composition (g/kg DM) of concentrate		

DM	880	880	
Starch	317	299	
Crude protein	190	190	
NDF	225	195	
ADF	97	98	
ADL	22	23	
Fat	56	56	
**Cow – Sunflower cake [46]**			
	Control diet	Experimental diet	
DMI (kg/day)	nr	nr	
Ingredient (kg/day per cow as fed)			
Maize silage	24	26	
Alfalfa hay	2	2	
Grass hay	2	2	
Whole cottonseeds	1	1	
Grain and protein mix	3.6	3.6	
Soybean meal	2	-	
Sunflower cake	-	2	
Ground maize	4.8	4.8	
Vitamin and mineral supplement	0.2	0.2	
Sodium bicarbonate	0.1	0.1	
Total soybean meal	2.5	0.5	
Total sunflower cake	-	2	
Chemical composition (% DM if not otherwise stated)			
DM (% weight)	55.1	57.4	
Crude protein	15.5	15.4	
NDF	36.7	39.4	
ADF	20.6	23.8	
Ether extract	3.7	4.4	
Starch	25.5	24.6	
Ash	5.7	5.8	
**Sheep – Sunflower meal [48]**			
	Control diet	Experimental diet	
DMI (kg/day)	1.88	1.89	1.94
Ingredient (% DM)			
Barley straw	30	30	30
Wheat bran	10	10	10
Barley grain	40.3	29.3	20.8
Soybean meal	18	9	-
Sunflower meal	-	20	37.5
Limestone	0.5	0.5	0.5
Dicalcium phosphate	0.5	0.5	0.5
Salt	0.6	0.6	0.6
Trace minerals + vitamin mixture	0.1	0.1	0.1
Chemical composition (% DM if not otherwise stated)			
Moisture (%)	8.3	7.9	7.7
Ash	8.4	8.4	7.1
Crude protein	16.3	16.5	16.4
Crude fibre	20.9	25.9	25.6
Ether extract	2.0	2.3	3.7
Nitrogen free extract	52.3	47	47.3
NDF	69.2	45.9	55.8
ADF	21.1	23.2	31.8
ME (MJ/kg)	9.7	9.2	8.8
**Sheep – Sunflower cake [69]**			
	Control diet	Experimental diet	
DMI (kg/day)	nr	nr	

<table>
<tr><td colspan="6">Ingredient (g/kg as fed) in concentrate</td></tr>
<tr><td colspan="2">Corn</td><td colspan="2">705</td><td colspan="2">678</td></tr>
<tr><td colspan="2">Barley</td><td colspan="2">100</td><td colspan="2">50</td></tr>
<tr><td colspan="2">Soybean meal</td><td colspan="2">165</td><td colspan="2">62</td></tr>
<tr><td colspan="2">Sunflower cake</td><td colspan="2">-</td><td colspan="2">180</td></tr>
<tr><td colspan="2">Mineral premix</td><td colspan="2">30</td><td colspan="2">30</td></tr>
<tr><td colspan="6">Chemical composition (g/kg DM if not otherwise stated) of concentrate</td></tr>
<tr><td colspan="2">DM</td><td colspan="2">897.5</td><td colspan="2">910.2</td></tr>
<tr><td colspan="2">Crude protein</td><td colspan="2">140.7</td><td colspan="2">152.9</td></tr>
<tr><td colspan="2">Ether extract</td><td colspan="2">29.9</td><td colspan="2">34.2</td></tr>
<tr><td colspan="2">Crude fiber</td><td colspan="2">32.7</td><td colspan="2">46.6</td></tr>
<tr><td colspan="2">Ash</td><td colspan="2">39.3</td><td colspan="2">47.6</td></tr>
<tr><td colspan="2">NDF (%)</td><td colspan="2">9.13</td><td colspan="2">12.10</td></tr>
<tr><td colspan="2">Non-fiber carbohydrates (%)</td><td colspan="2">69.88</td><td colspan="2">64.43</td></tr>
<tr><td colspan="2">NE_L (MJ/kg)</td><td colspan="2">7.71</td><td colspan="2">7.69</td></tr>
<tr><td colspan="6">Sheep – Sunflower cake [44]</td></tr>
<tr><td colspan="2"></td><td colspan="2">Control diet</td><td colspan="2">Experimental diet</td></tr>
<tr><td colspan="2"></td><td>Experiment I</td><td>Experiment II</td><td>Experiment I</td><td>Experiment II</td></tr>
<tr><td colspan="2">DMI (kg/day)</td><td>nr</td><td>nr</td><td>nr</td><td>nr</td></tr>
<tr><td colspan="6">Ingredient</td></tr>
<tr><td colspan="6">Soya was replaced with different amounts of sunflower oilcake (50% by weight of the total concentrate in Experiment I (indoor feeding), and 30% by weight of the total concentrate in Experiment II (outdoor grazing)) in the experimental concentrates.</td></tr>
<tr><td colspan="6">Chemical composition (% if not otherwise stated) of concentrate</td></tr>
<tr><td colspan="2">Crude protein</td><td>15.0</td><td>16.4</td><td>15.0</td><td>13.7</td></tr>
<tr><td colspan="2">Crude fat</td><td>5.0</td><td>5.2</td><td>11.0</td><td>6.7</td></tr>
<tr><td colspan="2">Energy (UFL)</td><td>1.1</td><td>1.0</td><td>1.1</td><td>1.3</td></tr>
<tr><td colspan="6">Sheep – Sunflower cake [70]</td></tr>
<tr><td colspan="2"></td><td colspan="2">Control diet</td><td colspan="2">Experimental diet</td></tr>
<tr><td rowspan="2">DMI (kg/day)</td><td>fescue hay</td><td colspan="2">1.86</td><td colspan="2">1.83</td></tr>
<tr><td>sainfoin hay</td><td colspan="2">2.70</td><td colspan="2">2.74</td></tr>
<tr><td colspan="6">Ingredient (g/kg DM) in concentrate</td></tr>
<tr><td colspan="2">Sunflower cake</td><td colspan="2">-</td><td colspan="2">560</td></tr>
<tr><td colspan="2">Soybean meal</td><td colspan="2">150</td><td colspan="2">-</td></tr>
<tr><td colspan="2">Barley</td><td colspan="2">160</td><td colspan="2">150</td></tr>
<tr><td colspan="2">Corn</td><td colspan="2">180</td><td colspan="2">210</td></tr>
<tr><td colspan="2">Oats</td><td colspan="2">200</td><td colspan="2">-</td></tr>
<tr><td colspan="2">Molasses</td><td colspan="2">50</td><td colspan="2">50</td></tr>
<tr><td colspan="2">DDGs</td><td colspan="2">150</td><td colspan="2">-</td></tr>
<tr><td colspan="2">Hydrogenated palm fat</td><td colspan="2">80</td><td colspan="2">-</td></tr>
<tr><td colspan="2">Vitamin-mineral premix</td><td colspan="2">30</td><td colspan="2">30</td></tr>
<tr><td colspan="6">Chemical composition (g/kg DM) of concentrate</td></tr>
<tr><td colspan="2">DM</td><td colspan="2">904</td><td colspan="2">902</td></tr>
<tr><td colspan="2">Organic matter</td><td colspan="2">847</td><td colspan="2">833</td></tr>
<tr><td colspan="2">Crude protein</td><td colspan="2">185</td><td colspan="2">180</td></tr>
<tr><td colspan="2">NDF</td><td colspan="2">201</td><td colspan="2">286</td></tr>
<tr><td colspan="2">ADF</td><td colspan="2">53</td><td colspan="2">200</td></tr>
<tr><td colspan="2">Fat</td><td colspan="2">107</td><td colspan="2">108</td></tr>
<tr><td colspan="2">Starch</td><td colspan="2">338</td><td colspan="2">267</td></tr>
<tr><td colspan="2">Feed Unit for Lactation</td><td colspan="2">1.1</td><td colspan="2">1.1</td></tr>
<tr><td colspan="6">Goat – Sunflower meal [71]</td></tr>
<tr><td colspan="2"></td><td colspan="2">Control diet</td><td colspan="2">Experimental diet</td></tr>
<tr><td colspan="2">DMI (kg/day)</td><td colspan="2">nr</td><td colspan="2">nr</td></tr>
<tr><td colspan="6">Ingredient (kg/day per goat as fed)</td></tr>
<tr><td colspan="2">Pasture grass</td><td colspan="2">6</td><td colspan="2">6</td></tr>
<tr><td colspan="2">Hay</td><td colspan="2">1</td><td colspan="2">1</td></tr>
<tr><td colspan="2">Oat meal</td><td colspan="2">1</td><td colspan="2">0.8</td></tr>
</table>

Sunflower meal	-	0.2

**Cow – Camelina seed cake* [54]**			
	Control diet	Experimental diet	
DMI (kg/day)	18.99	19.00	19.02
Ingredient (kg DM/cow)			
Alfalfa hay	2.55	2.55	2.55
Brewers grains	0.82	0.82	0.82
Fresh Sudan grass	9.76	9.76	9.76
Compound feed	5.85	5.87	5.89
Ingredient (%) in compound feed			
Corn	51.8	51.8	51.8
Wheat bran	12.9	12.9	12.9
Sunflower meal	31.0	15.5	-
Camelina meal	-	15.5	31.0
Calcium carbonate	2.3	2.3	2.3
Salt	1.0	1.0	1.0
Vitamin-mineral premix	1.0	1.0	1.0

**Cow – Camelina seed cake* [53]**		
	Control diet	Experimental diet
DMI (kg/day)	21.0	19.8
Ingredient (%)		
Corn silage	57.8	57.8
Soybean meal	10.2	8.9
Energy concentrate (barley, wheat, beet pulp, wheat bran, molasses, $CaCO_3$, $NaHCO_3$, and NaCl)	30.2	22.5
Camelina seed cake*	-	9.5
Urea	0.6	0.1
Minerals	1.3	1.1
Energy and protein balances		
NE_L (Mcal/day)	31.2	30.1
PDIN (protein digested in the small intestine (PDI) supplied by rumen-undegraded dietary protein and by microbial protein from rumen-degraded OM) (g/day)	2075	2065
PDIE (PDI supplied by rumen-undegraded dietary protein and by microbial protein from rumen fermented OM) (g/day)	2020	1916
Energy balance (Mcal/day)	0.3	6.3
PDI balance (g/day)	61.0	103.0

**Cow – Camelina seed expeller [28]**		
	Control diet	Experimental diet
DMI (kg/day)	23.7	24.4
Ingredient (g/kg DM)		
Grass hay, chopped	50	50
Grass silage	280	280
Corn silage	150	150
Rolled barley	295	370
Corn gluten meal	25	35
Corn DDGS	180	-
Camelina expeller	-	95
Calcium carbonate	6	6
Iodized salt	2	2
Magnesium oxide	2	2
Mineral and vitamin premix	10	10
Chemical composition (g/kg DM if not otherwise stated)		
DM (g/kg as fed)	538	538

ME (Mcal/kg DM)	2.43		2.44	
Crude protein	147		150	
ADF	237		239	
NDF	381		395	
Ether extract	41		42	
**Cow – Camelina seed expeller [78]**				
	Control diet		Experimental diet	
DMI (kg/day)	23.3		22.7	
Ingredient (% on an air-dry basis) in concentrate				
Barley	20.0		20.0	
Wheat	20.0		20.0	
Camelina expeller	-		20.0	
Molassed sugar-beet pulp	20.0		20.0	
Cereal bran	11.2		10.6	
Sugar-beet molasses	6.0		6.0	
Rapeseed oil	-		0.6	
Calcium carbonate	1.4		1.4	
Sodium chloride	0.7		0.7	
Magnesium oxide	0.3		0.3	
Mineral premix	0.2		0.2	
Vitamin premix	0.2		0.2	
Chemical composition (% DM if not otherwise stated) of concentrate				
DM (% as fed)	87.4		88.2	
Organic matter	93.0		91.2	
Crude protein	16.5		16.4	
NDF	22.5		22.7	
Fatty acids	3.14		4.90	
**Cow – Camelina seed expeller [56]**				
	Control diet	Experimental diet		
DMI (kg/day)	26.9	27.1	26.9	26.7
Ingredient (% DM)				
Barley silage	31.6	31.6	31.6	31.6
Alfalfa hay	14.0	14.0	14.0	14.0
Barley grain	24.2	24.2	24.2	24.2
Camelina expeller meal	-	5.02	7.51	10.0
Canola meal	17.6	12.5	10.0	7.5
Corn gluten meal	0.04	0.04	0.04	0.04
Soybean meal	0.04	0.04	0.04	0.04
Soybean hulls	4.04	4.04	4.04	4.04
Cottonseed hulls	3.51	3.51	3.51	3.51
Oat hulls	1.4	1.4	1.4	1.4
Molasses (dried)	1.05	1.05	1.05	1.05
Sodium bicarbonate	0.78	0.78	0.78	0.78
Limestone	0.18	0.18	0.18	0.18
Dynamate	0.14	0.14	0.14	0.14
Mineral-vitamin premix	1.4	1.4	1.4	1.4
Chemical composition (% DM if not otherwise stated)				
DM (%)	60.4	61.4	61.0	61.2
Crude protein	18.1	17.8	17.9	17.6
NDF	37.6	37.2	37.6	37.4
ADF	24.6	25.2	25.0	25.1
Starch	21.8	20.8	20.4	20.6
Crude fat	2.02	2.39	2.65	2.78
Ash	8.48	8.37	9.18	8.48
NE_L (Mcal/kg)	1.56	1.56	1.58	1.59
**Cow – Camelina seed cake [77]**				
The diets were based on *ad libitum* grass silage, mineral feed, 8 kg of concentrates prepared from barley and oat				

meal, soybean meal and cold pressed oil cakes as fat sources (0.5 kg crude fat per animal per day): gold of pleasure (Camelina sativa) cake and soybean meal as a non-fat control variant.			
**Sheep – Camelina seed cake [55]**			
	Control diet	Experimental diet	
DMI (kg/day)	nr	nr	nr
Ingredient (g/kg DM) in concentrate			
Wheat	700	700	700
Wheat bran	80	80	80
Rapeseed meal	200	100	-
Camelina sativa cake	0	100	200
Mineral and vitamins	20	20	20
Chemical composition (g/kg DM) of concentrate			
Organic matter	931	933	941
Crude protein	180	176	174
Crude fat	24	38	51
ADF	74	83	73
NDF	159	163	172
**Goat – Camelina seed cake [52]**			
	Control diet	Experimental diet	
DMI (kg/day)	nr	nr	
Ingredient (g/kg DM) in concentrate			
Wheat	700	700	
Wheat bran	80	80	
Rapeseed extracted meal	200	80	
Camelina sativa cake	-	120	
Minerals and vitamins	20	20	
Chemical composition (g/kg DM) of concentrate			
DM	88.49	91.63	
Organic matter	83.25	87.50	
Crude ash	5.24	4.13	
Crude protein	17.20	17.95	
Crude fibre	5.13	4.83	
Crude fat	3.46	4.45	
NDF	15.39	15.71	
**Cow – Linseed cake [74]**			
	Control diet	Experimental diet	
DMI (kg/day)	nr	nr	
Ingredient (kg/day)			
Grass silage	24.00	24.00	
Alfalfa silage	71.00	71.00	
Corn silage	3.50	3.50	
Pasture ground chalk	0.10	0.10	
Vitamin mix	0.14	0.14	
Salt	0.05	0.05	
Magnesium oxide	0.05	0.05	
Linseed cake	-	0.30	
Chemical composition (% DM if not otherwise stated)			
DM (%)	57.50	58.20	
Ash	4.20	4.45	
Crude protein	7.50	7.75	
ADF	27.90	28.70	
NDF	33.70	34.60	
Crude fibre	4.36	4.56	
UFL per kg of DM	1.10	1.16	
**Cow – Linseed meal [47]**			
	Control diet	Experimental diet	
DMI (kg/day)	15.58	15.67	

Ingredient (% DM) in concentrate		
Corn meal	12.00	12.00
Flaxseed meal	-	16.00
Soybean meal	11.00	-
Sunflower meal	5.00	-
Minerals and vitamin premix	2.00	2.00
Chemical composition (% DM if not otherwise stated) of concentrate		
DM (% of fresh matter)	94.79	92.92
Crude protein	27.11	24.98
NDF	13.99	17.22
ADF	7.97	8.92
NDIN	0.60	0.30
ADIN	0.14	0.10
Crude fat	5.19	1.88
Ash	7.71	6.76
Lignin	3.89	3.33
Starch	31.14	31.71
**Cow – Linseed meal [64]**		
	Control diet	Experimental diet
DMI (kg/day)	31.5	32.6
Ingredient (g/kg DM)		
Grass, silage	301	300
Corn, silage	303	301
Corn, grain (cracked)	185	183
Beet pulp	66	28
Soybean, meal (480 g/kg CP, solvent)	88	-
Flax, meal (363 g/kg CP)	-	124
Top supplement (canola meal, corn gluten meal, soybean meal, brewer's corn)	40	46
Minerals and vitamins	13	14
Ca carbonate	4	4
Chemical composition (g/kg DM if not otherwise stated)		
DM (g/kg)	530	529
Crude protein	175	181
Fat	31.3	31.7
aNDF	315	319
ADF	224	222
NE_L (MJ/kg DM)	6.9	6.9
**Cow – Linseed cake* [60]**		
	Control diet	Experimental diet
DMI (kg/day)	12.98	13.50
Ingredient in concentrate		
Crushed corn	40	38
Soybean meal	6.5	3
Wheat brain	24	22
Sunflower meal	23	21.5
Linseed meal	-	9
Molasses	3	3
Na-Cl salt	1	1
Limestone	2	2
Minerals mix	0.5	0.5
Chemical composition (% DM if not otherwise stated) of concentrate		
DM (%)	88.02	88.02
Crude protein	16.11	16.75
Crude fibre	9.87	9.69
Ether extract	3.04	3.25
NFE	64.02	63.28

Ash	6.96	7.03
Organic matter (%)	93.04	92.97
Chemical composition (%)		
DM (%)	90.66	89.62
Crude protein	13.03	13.50
Ether extract	2.65	3.40
Crude fibre	22.15	21.68
NFE	47.14	49.30
**Cow – Linseed meal [62]**		
	Control diet	Experimental diet
DMI (kg/day)	17.7	17.6
Ingredient (%) in concentrate		
Corn	67.5	67.5
Canola meal	24.6	-
Linseed meal	-	24.6
Molasses	3.5	3.5
Iodized salt	1.37	1.37
Limestone	0.56	0.56
Trace-mineralized salt	2.35	2.35
Vitamin mix	0.07	0.07
Vitamin D_3	0.01	0.01
Chemical composition (% DM) of concentrate		
Organic matter	91.2	92.0
NDF	19.0	18.4
ADF	6.7	6.6
Cellulose	4.6	4.5
Lignin	3.4	2.1
Crude protein	19.7	19.6
Starch	39.6	42.2
Chemical composition (% DM if not otherwise stated)		
DM (%)	43.6	49.1
Organic matter	89.7	90.7
NDF	42.8	41.5
ADF	23.3	22.5
Cellulose	18.1	18.8
Lignin	5.2	3.7
Crude protein	15.7	14.5
Ammonia N	1.2	1.2

**Cow – Linseed expeller [75]**				
	Control diet	Experimental diet		
DMI (kg/day)	21.0	20.3	21.1	22.0
Ingredient (g/100 g DM)				
Maize silage	292	291	290	290
Grass silage	315	316	317	314
Ground maize grain	211	202	192	189
Soybean meal	108	74.5	41.4	20.4
Flax meal	-	47.9	95.3	141
Top supplement (rapeseed meal, maize gluten meal, soyabean meal, brewer's maize)	17.4	17.4	17.9	8.5
Beet pulp	34.3	29.6	25.0	16.1
Calcium carbonate	5.5	5.5	5.4	5.5
Minerals and vitamins	16.4	16.3	16.3	15.5
Chemical composition (g/100 g DM if not otherwise stated)				
DM (g/100 g diet)	37.7	37.9	37.6	38.1
Crude protein	17.0	17.4	17.6	17.9
Fat	2.44	2.41	2.34	2.41
ADF	18.3	18.5	19.2	19.3

NDF	28.4	28.6	29.5	29.6
NE$_L$ (kJ/g DM)	6.65	6.61	6.61	6.61
**Cow – Linseed meal [76]**				
	Control diet		Experimental diet	
DMI (kg/day)	21.4		20.2	
Ingredient (% DM)				
Timothy silage	7.7		9.1	
Alfalfa-timothy silage	25.9		27.4	
Corn silage	9.2		10.5	
Barley	28.6		25.0	
Corn grain	11.2		8.7	
Protein supplement	16.6		9.1	
Flaxseed meal	-		9.4	
Sodium bicarbonate	0.8		0.8	
Chemical composition (% DM if not otherwise stated)				
DM (%)	47.9		45.5	
NE$_L$ (Mcal/kg DM)	1.61		1.60	
Crude protein	18.3		18.6	
NDF	36.5		37.4	
ADF	22.4		23.2	
Total fatty acids	0.9		1.4	
**Cow – Linseed cake [59]**				
	Control diet	Experimental diet		
DMI (kg/day)	14.26	14.53	13.81	14.08
Feed intake (kg DM/day)				
Silage	9.06	9.33	8.71	9.17
Barley and oats mixture	3.83	3.83	3.76	3.62
Rapeseed cake	1.37	0.91	0.45	-
Linseed cake	-	0.46	0.89	1.29
Nutrient intake (g/day if not otherwise stated)				
Organic matter (kg/day)	13.38	13.63	12.97	13.21
Crude protein	2461	2530	2418	2445
Ether extract (total)	743	769	759	786
Ether extract (from rapeseed cake)	160	107	52	0
Ether extract from linseed cake)	0	73	143	207
ME (MJ/day)	166.4	168.9	162.0	165.9
**Cow – Linseed cake [58]**				
	Control diet		Experimental diet	
DMI (kg/day)	16.18		17.07	
Ingredient (% DM) in concentrate				
Rapeseed cake	26.51		-	
Linseed cake	-		21.31	
Triticale	28.91		33.71	
Soybean meal	25.03		25.03	
Ketomix E18	11.89		11.89	
Chalk	1.83		1.83	
Co-bind A-Z	0.57		0.57	
Sodium bicarbonate	2.40		2.40	
Witamix KW	2.86		2.86	
**Cow – Linseed cake [77]**				
The diets were based on *ad libitum* grass silage, mineral feed, 8 kg of concentrates prepared from barley and oat meal, soybean meal and cold pressed oil cakes as fat sources (0.5 kg crude fat per animal per day): linseed cake and soybean meal as a non-fat control variant.				
**Goat – Linseed cake extruded [61]**				
	Control diet	Experimental diet		
DMI (kg/day)	nr	nr	nr	
Ingredient (%)				

Commercial mix (alfalfa hay, corn meal, soybean hull, beet pulp, sunflower meal, wheat bran, flaked corn, barley, soybean meal, cane molasses, vitamins and minerals supplement)	74	74	74
Concentrate (corn, barley, beet pulp)	16	16	16
Extruded linseed cake	-	5	10
Peas	10	5	-
Chemical composition (% DM if not otherwise stated)			
DM (%)	88.8	88.9	89.3
Crude protein	17.5	17.9	18.3
NDF	38.5	38.6	38.7
ADF	21.8	22.1	22.5
ADL	4.1	4.5	4.9
Ash	7.7	7.8	8.0
Lipid extract	2.4	3.1	3.9

**Goat – Linseed cake [57]**		
	Control diet	Experimental diet
DMI (kg/day)	nr	nr
Ingredient (% DM)		
Corn silage	29.7	30.7
Meadow hay	29.2	30.1
Oats (grain)	18.7	19.3
Triticale (grain)	11.2	-
Extracted rapeseed meal	11.2	-
Linseed cake	-	19.9
Chemical composition (g/kg DM if not otherwise stated)		
NE_L (MJ)	6.29	6.25
Crude protein	142.4	147.5
PDI	102.6	93.8
Crude fibre	184.4	216.2
NDF	387.4	535.7
ADF	213.7	290.8
ADL	3.2	4.7
Ether extract	29.6	35.7

DM – dry matter; DMI – dry matter intake; NDF - neutral detergent fiber; ADF - acid detergent fiber; ADL - acid detergent lignin; ME - metabolisable energy; NE_L - net energy for lactation; DDGS - dried distillers grains with solubles; * - estimated by the authors based on the content of fat in byproduct
